# Hepatitis E Virus Detection in Liver Tissue from Patients with Suspected Drug-Induced Liver Injury

**DOI:** 10.3389/fmed.2015.00020

**Published:** 2015-03-30

**Authors:** Obinna Chijioke, Marion Bawohl, Erik Springer, Achim Weber

**Affiliations:** ^1^Institute of Surgical Pathology, University Hospital Zurich, University of Zurich, Zurich, Switzerland; ^2^Institute of Pathology, Johannes Gutenberg University Mainz, Mainz, Germany

**Keywords:** liver, hepatitis, hepatitis E virus infection, drug-induced liver injury, molecular testing

## Abstract

Hepatitis E virus (HEV) infection is increasingly recognized as a cause of acute hepatitis in the industrialized world. We aimed to determine the frequency of acute HEV infection in cases of suspected drug-induced liver injury (DILI), mainly a diagnosis of exclusion. To this aim, formalin-fixed paraffin-embedded (FFPE) liver tissues of all cases routinely processed in our institute during a 2 1/2 years period in which DILI was among the differential diagnoses (157 liver biopsies, 1 liver explant) were subjected to semi-nested RT-PCR for the detection of HEV RNA. Histopathology was re-evaluated on all cases tested positive. HEV RNA was detectable in 3 of 158 cases (2%) tested, comprising autochthonic as well as travel-related infections with genotypes 1, 3, and 4 each found once, respectively. Histopathologic findings comprised one case with subtotal hepatic necrosis and two cases of acute (cholestatic) hepatitis not distinguishable from acute hepatitis of other etiology. Thus, the overall frequency of acute HEV infection as determined by detection of HEV RNA in liver tissue is substantially increased in patients with suspected DILI compared to the healthy population, emphasizing the need to actively look for HEV infection in cases of suspected DILI. Molecular testing for HEV RNA in routinely processed FFPE liver tissues can be applied to cases with undetermined HEV status.

## Introduction

Hepatitis E virus (HEV) has long been known to cause epidemic infection leading to acute liver disease mostly in the developing world without affecting industrialized countries. However, more recent data have established increased rates of autochthonous infection in some European countries (1, 2) with the infection considered to be zoonotic and food-borne instead of waterborne as in developing countries (3, 4). HEV RNA is reported to be detectable in <0.1% of the healthy population in industrialized countries, e.g., in 0.037% of blood donors in the Netherlands (5) and 0.08% of blood donors in Germany (6), although seroprevalence might be much higher (5, 7). Symptomatic infection with HEV can cause acute liver disease indistinguishable from hepatitis A and might even be the more common infectious agent causing acute hepatitis (3, 8, 9). Still, testing for HEV in cases of acute hepatitis is not done on a regular basis, due in part to uncertainties in the consistency of serological assays based on the detection of HEV-specific antibodies (7, 10).

Drug-induced liver injury (DILI) is a diagnosis of exclusion and recent studies have shown high incidences in industrialized countries (11, 12). DILI presents with a wide spectrum of histological patterns including changes mimicking acute hepatitis (13), and in the absence of conclusive testing for HEV, might mask underlying hepatic injury primarily due to infection with HEV. Indeed, HEV infection has been documented in cases of suspected DILI serologically via detection of viral RNA in serum (14, 15) as well as in liver tissue from hepatitis cases of clinically unexplained origin (16). This study was designed to determine the presence of HEV infection in liver tissue and its frequency in cases in which DILI was among the differential diagnoses.

## Materials and Methods

### Patient samples and study design

One hundred and fifty seven liver biopsies and one liver explant obtained from individual patients at the University Hospital Zurich, Switzerland during 2011, 2012, and first half of 2013 were retrospectively included in our study. All patients had a suspected DILI based on either clinical and/or histological findings. Patients with previous liver transplantation were excluded from the study. This retrospective study was reviewed and approved by the internal review board of the University Hospital Zurich and the Cantonal Ethics Committee of Zurich, Switzerland (KEK-ZH-Nr. 2013-0504).

### RNA extraction, RT-PCR, and sequencing

RNA from formalin-fixed paraffin-embedded (FFPE) tissue was isolated using Trizol^®^ LS (Life Technologies). In brief, FFPE tissue was cut, incubated in 0.02M Tris/0.02 EDTA/1% SDS at 95°C for 10 min, centrifuged at 4°C, and solid paraffin removed. Tissue was digested with proteinase K (55°C for 48–72 h), Trizol LS added to the solution, and homogenized using shredding columns (Qiagen). After addition of chloroform, the flow-through was further subjected to centrifugation and the aqueous upper part removed to precipitate total RNA with 2-propanol. One microgram of total RNA was reverse transcribed (iScript™ cDNA synthesis kit, Bio-Rad) and resulting cDNA tested for the presence of HEV using a semi-nested PCR approach targeting a HEV-specific gene region. Oligo nucleotide primers for first round amplification were: F: 5-TCGGGTGGAATGAATAACATGT-3 and R1: 5-GGTTGGTTGGATGAATATAAGGGG -3 resulting in a 232 bp amplicon. Oligo nucleotide primers for second round amplification were: F: 5-TCGGGTGGAATGAATAACATGT-3 and R2: 5-CGGTCACCCCAGAAACCAC-3 resulting in a 185 bp amplicon. Oligo nucleotide primer sequences are based on a whole-genome alignment of 165 HEV-strains and conserved regions therein corresponding to nucleotides of HEV reference sequence HQ634346.1: F: nt 5135-5156, R1: nt 5366-5388; R2: nt5319-5301, respectively.

RT-PCR for β-actin and a second housekeeping gene (GAPDH) by real time RT-PCR was performed for all samples analyzed. PCR products were sequenced by direct Sanger sequencing to confirm specificity of the amplicon. Sequences were aligned to publicly accessible HEV reference sequences to determine HEV genotype using the BLAST program (http://www.ncbi.nlm.nih.gov).

The detection of HEV RNA was confirmed by parallel or retrospective serum testing in the Swiss reference laboratory for HEV testing (Prof. Darius Moradpour, Gastroenterology and Hepatology Division, CHUV, Lausanne).

### Histology

Histological evaluation as provided by initial surgical pathological report was recorded including extent of inflammation, fibrosis, cholestasis, steatosis, and architectural alterations. Histological reevaluation was performed on all HEV RNA positive cases.

## Results

### Characteristics of patients with suspected drug-induced liver injury

In total, 157 cases in which DILI was among the differential diagnoses and patients underwent liver biopsy and 1 liver explant from a patient with suspected DILI from January 2011 through June 2013 were identified and liver tissue specimen analyzed for the presence of HEV RNA. Histological features of necroinflammatory activity and fibrosis according to the classification of Batts and Ludwig (17), steatosis and cholestasis as well as gender and age of patients are summarized in Table 1. Female and male patients were equally distributed in this cohort (females: 51%, males: 49%) and more than half belonged to the group of patients 30–60 years of age (Table 1). No specific medication appeared to be disproportionally frequently taken, except for amoxicillin/clavulanic acid (5 cases) and azathioprine (3 cases), instead no other single medication was implicated in more than one case and medication covered a wide range of drugs (data not shown). Necroinflammatory activity was mostly present (absent in only 4%) and varying degrees of fibrosis were common (67%). Cholestasis was noted in one-third of cases, mostly canalicular (21%) or hepatocellular (11%) and the majority of cases had absence of steatosis (59%).

**Table 1:** **Characteristics of study patients with suspected drug-induced liver injury**.

	*N* (%)
Female	81 (51.3)
Age (years)	
<30	22 (13.9)
30–60	91 (57.6)
>60	45 (28.5)
Necroinflammatory activity	
Grade 0	6 (3.8)
Grade 1	58 (36.7)
Grade 2	71 (44.9)
Grade 3	20 (12.7)
Grade 4	3 (1.9)
Fibrosis	
Grade 0	53 (33.5)
Grade 1	58 (36.7)
Grade 2	20 (12.7)
Grade 3	18 (11.4)
Grade 4	9 (5.7)
Cholestasis	
Hepatocellular	18 (11.4)
Canalicular	33 (20.9)
Ductal	5 (3.2)
Absent	102 (64.6)

### Detection of viral RNA in liver tissues

An initial comparison of already established (18) and newly designed primer sets targeting well conserved viral sequences in three different semi-nested RT-PCR assays (data not shown) led to the identification of the most reliable protocol (oligos F, R1, and R2, see above). Using this protocol throughout the study, the overall frequency of HEV RNA detection in our study cohort was 2% (3/158). However, limitations inherent to RNA detection in FFPE were found when repetitive or parallel testing of individual cases was performed. HEV RNA was detectable in only 3 out of 4 different paraffin blocks of the hepatectomy specimen from case number 1 that presented with fulminant acute hepatitis and later underwent liver transplantation. Furthermore, case number 3 was tested positive in only 2 out of 6 runs. In contrast, case number 2 was always tested positive. Also, quality testing of RNA/cDNA with β-actin as an internal control gave positive results in only 151 out of 155 negative cases and analysis of a second housekeeping gene (GAPDH) via real time RT-PCR showed amplification mostly in late cycles (>30 cycles, Figures S1A–C in Supplementary Material and data not shown). Sequencing of positive cases confirmed the specificity of the amplification (Figures S1D–F in Supplementary Material).

### Clinico-pathological features of HEV RNA positive cases

**Figure 1:**
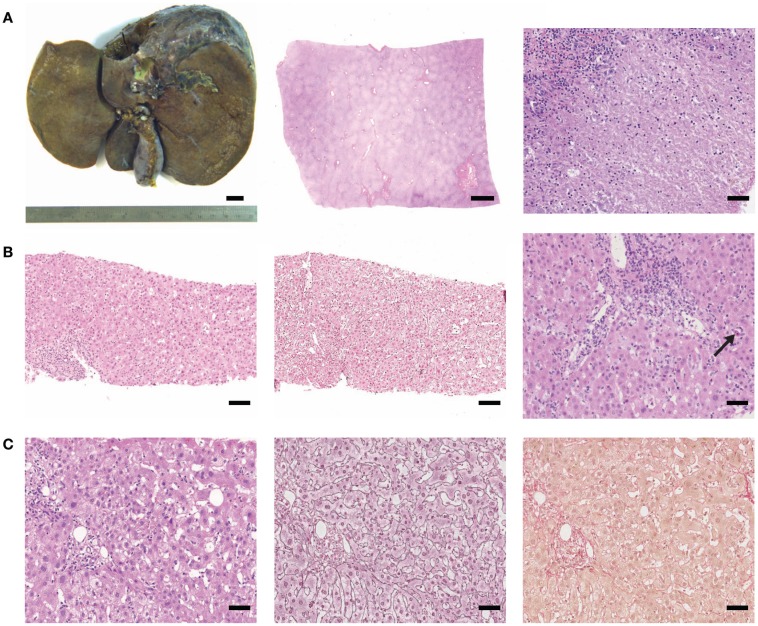
**(Histo)-pathological findings of cases tested positive for HEV RNA**. **(A)** Case 1: macroscopy of liver explant (left picture, bar represents 1 cm), subtotal necrosis (low magnification, middle micrograph, scale bar: 2 mm), and lobular inflammation with confluent necrosis (high magnification, right micrograph, scale bar: 50 μm). **(B)** Case 2: pronounced necro-inflammatory activity (H&E and silver reticulin stain, left and middle micrograph, respectively, scale bar: 100 μm). Moderate portal and lobular inflammation with spotty necrosis (arrow; right micrograph, scale bar 50 μm). **(C)** Case 3: mild inflammation (H&E, silver reticulin stain and sirius red, from left to right, scale bar: 50 μm).

Travel-associated HEV infection (genotype 1) was found in case number 1. This non-pregnant, 26 years old female patient reported traveling to India with one episode of fever (39°C) and recurrent epigastric pain during her stay. The epigastric pain persisted after her return and she developed emesis and nausea as well as generalized icterus shortly before hospitalization. Liver transplantation was performed due to fulminant liver failure. In the initial histopathological report of the explanted liver (Figure 1A for macroscopy and histology), the differential diagnoses of either an underlying infectious or toxic (drug-induced) etiology were discussed. However, testing for HEV RNA in autologous liver tissue (this study) and serum was positive. Already 1 week after liver transplantation, serum was negative for HEV RNA by RT-PCR. No further biopsy was taken afterward.

Case number 2 had a liver biopsy (Figure 1B) due to presentation with otherwise unexplained icterus for 2 weeks and elevated transaminases but low immunoglobulins. Acute cholestatic hepatitis was confirmed histologically (Figure 1B) with suspected toxic (drug-induced) liver injury and retrospectively, HEV RNA was detected in the liver biopsy (genotype 3).

In case number 3, liver failure with icterus and cholestatic hepatitis was reported clinically due to suspected DILI after intake of statins, however, intake was stopped 2 weeks before presentation. Autoimmune markers were negative and total serum immunoglobulin was not elevated. This patient also reported traveling to Corsica (mediterranean sea) 1 month before falling sick. The patient had a protracted clinical course with reconvalescence only after 9 months. HEV RNA (genotype 4) was retrospectively detected in liver tissue (this study) and also in stored serum taken during acute illness. Liver histology of case number 3 is shown in Figure 1C.

None of the three study patients revealed significant liver fibrosis. A summary of characteristics of HEV RNA positive cases including detailed histological findings is presented in Table 2.

**Table 2:** **Characteristics of HEV RNA positive cases**.

Case #	Case No. 1	Case No. 2	Case No. 3
Age	26	36	59
Sex	F	M	M
HEV genotype	1	3	4
HEV RNA in serum	Positive	Not available	Positive
	Traveled to India 1 month before presentation; acute liver failure (ALT > 8000 U/l, AST > 5000 U/l)	Autochthonous infection with no travel history; icteric, elevated aminotransferases (> 5 × ULN)	Statin intake stopped 2 weeks before presentation icteric, cholestatic hepatitis (ALT 550 U/l, AST 170 U/l)
Interface hepatitis (0–4)	2	3	1
Lobular inflammation (0–4)	3	2	1
Portal inflammation (0–4)	3	3	1
Focal necrosis (0–4)	4	1	1
Confluent necrosis (0–6)	6	0	0
Hepatocyte rosettes	Yes	No	No
Lobular disarray	Yes	Yes	Yes
Plasma cells	Yes	Yes	Yes
Eosinophils	None	Few	Few
Neutrophils	Isolated	None	None
Fibrosis stage (0–6)	0	0	0
Cholestasis grade (0–3)	3	1	2
Hepatocellular cholestasis	Yes	Yes	Yes
Canalicular cholestasis	No	No	Yes
Ductal cholestasis	No	No	No
Ductal paucity			
None	x	x	x
Mild			
Moderate to marked			
Ductal reaction	No	Mild	Moderate

## Discussion

In this study on a cohort of 158 patients, we found HEV RNA with a frequency of 2% in liver tissues from patients in which DILI was among the differential diagnoses. Compared to findings based on sera testing for HEV RNA, this rate is remarkably similar to data from an US American study, in which HEV RNA was detected in 4 out of 318 (1.3%) sera from patients with suspected DILI (15), but lower compared to a smaller British DILI cohort in which 6 out of 28 patients (21%) with criterion-referenced DILI had autochthonous HEV infection (14). In a recent report, real time RT-PCR performed on RNA extracted from liver tissue in cases of acute hepatitis of clinically unexplained origin, 7 out of 221 cases examined were positive for HEV and 4 out of these 7 positive cases were sequenced as being genotype 3 HEV (16). Taken into account that not all HEV-positive cases of our study cohort were consistently tested positive in repetitive or parallel testing – performed due to limited RNA-quality – it is conceivable that the actual rate of HEV infection in DILI cases might even be higher. Taken together, accumulating evidence from our and similar studies points to an underestimated role of HEV infection in industrialized countries as the culprit for acute hepatitis other than drug-induced or otherwise virally precipitated.

In line with a previous report (19), pathological analysis of the cases of this study did not reveal any histopathological findings specific or even highly characteristic for HEV infection, which could be helpful for differential diagnosis against DILI-related changes. Therefore, we suggest testing for HEV RNA in cases of suspected DILI, which is also possible on liver tissue as reported here and by others (16). RT-PCR for HEV can be expected to be the more reliable method when compared to antibody-based serological testing in which false positive as well as false negative results are a major concern (7, 10). However, we would like to caution that the quality of cDNA obtained from RNA isolated from paraffin-embedded liver tissue is crucial, but sometimes limited and might therefore lead to false negative results. Therefore, optimization of protocols that increase the quality of RNA extracted from FFPE liver tissue is needed.

Taken together, we, here, report for the first time the detection of HEV infection directly from liver tissue in a substantial cohort of patients with suspected DILI. In line with previous data based on sera testing (14, 15), we find an increased frequency of HEV RNA in patients with suspected DILI. Since histopathological findings of HEV are overlapping with patterns found in DILI, we suggested performing HEV testing in all cases of suspected DILI which is also possible from FFPE liver tissues.

## Conflict of Interest Statement

The authors declare that the research was conducted in the absence of any commercial or financial relationships that could be construed as a potential conflict of interest.

## Supplementary Material

The Supplementary Material for this article can be found online at http://journal.frontiersin.org/article/10.3389/fmed.2015.00020
