# Supplementary material for: Hepatitis E Virus Detection in Liver Tissue from Patients with Suspected Drug-Induced Liver Injury
Source: Front Med (Lausanne). 2015 Mar 30;2:20. doi: 10.3389/fmed.2015.00020 (PMC4378310; doi:10.3389/fmed.2015.00020)
Supplement: Supplementary file 1 [file presentation_1.pdf]

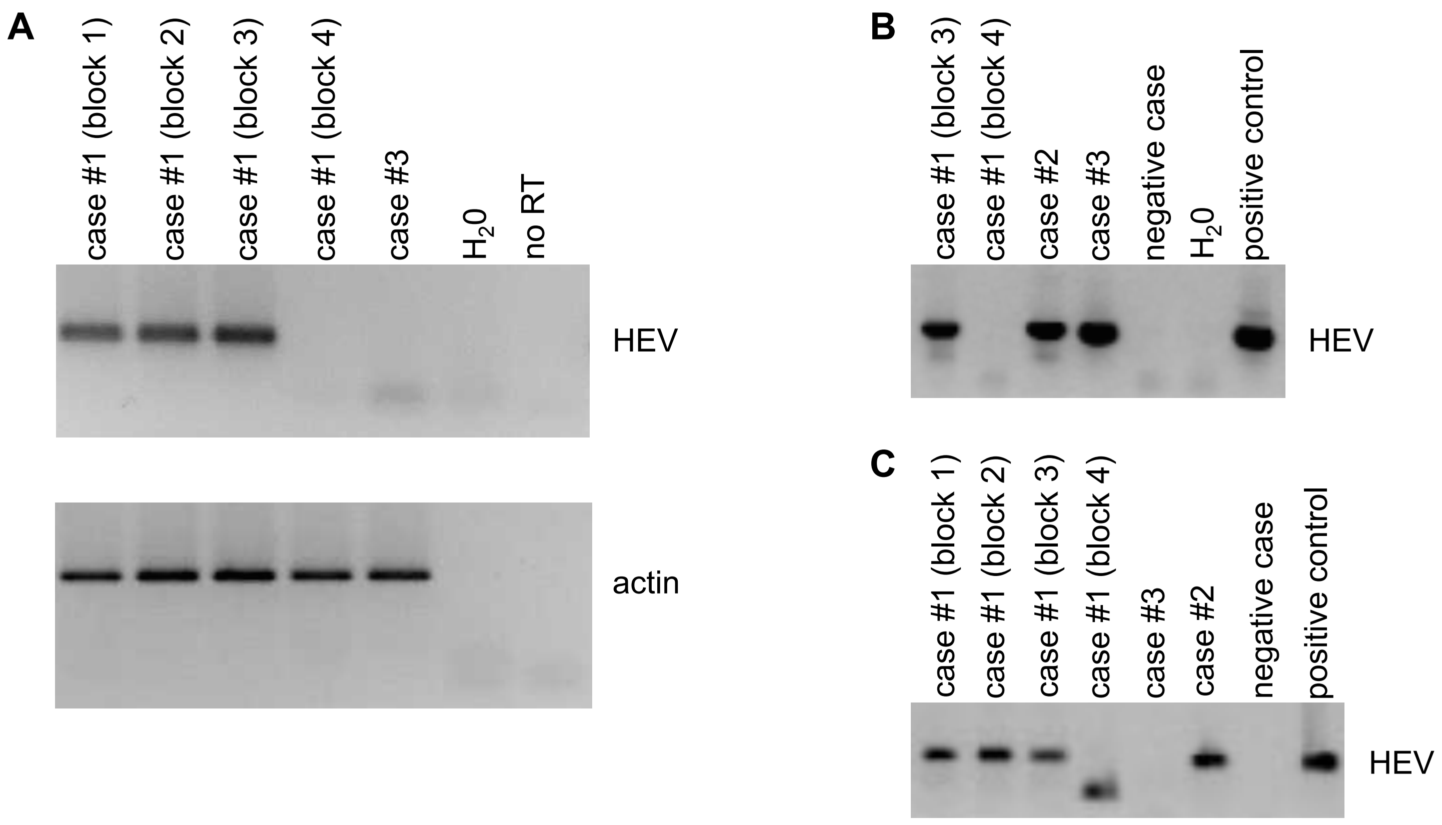
A
case #1 (block 1)
case #1 (block 2)
case #1 (block 3)
case #1 (block 4)
case #3
H20
no RT
HEV
actin
B
case #1 (block 3)
case #1 (block 4)
case #2
case #3
negative case
H20
positive control
HEV
C
case #1 (block 1)
case #1 (block 2)
case #1 (block 3)
case #1 (block 4)
case #3
case #2
negative case
positive control
HEV

D

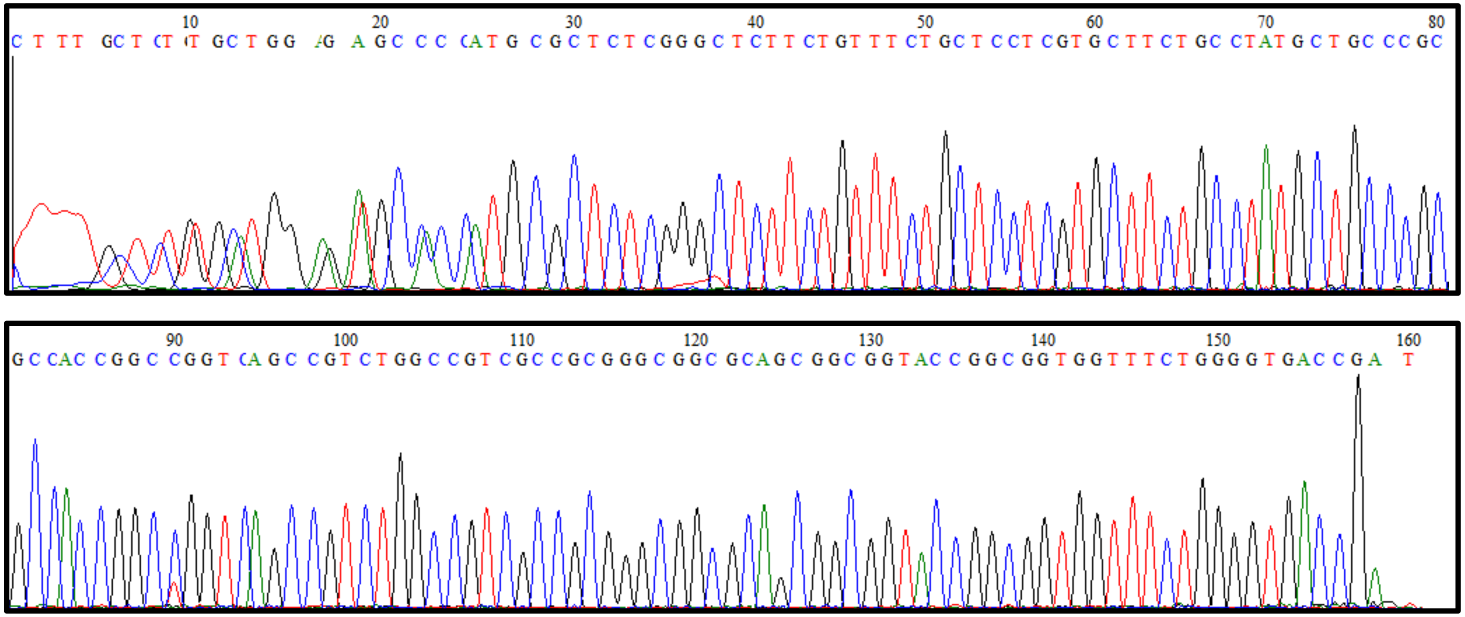

E

Download GenBank Graphics

Hepatitis E virus genomic RNA, complete genome, genotype 4, isolate: HE-JF3

Sequence ID: dbj|AB220971.1| **Length:** 7262 **Number of Matches:** 1

Range 1: 5138 to 5321 GenBank Graphics   Next Match Previous Match

Score	Expect	Identities	Gaps	Strand
329 bits(178)	7e-87	182/184(99%)	0/184(0%)	Plus/Plus

```
Query  2     CGGGTGGAATGAATAACATGTTCTTTTGCTCTGTGCATGGAGATGCCACCATGCGCTCTC  61
             ||||||||||||||||||||||||||||||||||||||||||||||||||||||||||||
Sbjct  5138  CGGGTGGAATGAATAACATGTTCTTTTGCTCTGTGCATGGAGATGCCACCATGCGCTCTC  5197

Query  62    GGGCTCTTCTGTTTCTGCTCCTCGTGCTTCTGCCTATGCTGCCCGCGCCACCGGCCGGTC  121
             |||||||||||||||||||| |||||||||||||||||||||||||||||||||||||||
Sbjct  5198  GGGCTCTTCTGTTTCTGCTCTTCGTGCTTCTGCCTATGCTGCCCGCGCCACCGGCCGGTC  5257

Query  122   AGCCGTCTGGCCGTCGCCGCGGGCGGCGCAGCGGCGGTACCGGCGGTGGTTTCTGGGGTG  181
             |||||||||||||||||||||||||||||||||||||| |||||||||||||||||||||
Sbjct  5258  AGCCGTCTGGCCGTCGCCGCGGGCGGCGCAGCGGCGGTGCCGGCGGTGGTTTCTGGGGTG  5317

Query  182   ACCG  185
             ||||
Sbjct  5318  ACCG  5321
```

F

```
            10        20        30        40        50        60        70        80        90
pt2  TCGGGTGGAATGAATAACATGTTTTATGCAGTGCCCATGGGATCACCATGTGCCCTCGGGCTGTTCTGTTGTTGCTCTTCGTGCTTTTGCCTATG
pt3  .......................C.T.TGCTCTGTGCAT...GATG.CACCATG.G.TCT.G.GCTCT.CTG.TTCTGCTCCT.G.GCTT..GCC

        100       110       120       130       140       150       160       170       180       190
     CTGCCCGCGCCACCGGCCGGCCAGCCGACTGGCCGCCGTCGTGGGCGGCGCAGCGGCGGTACCGGCAGTGGTTTCTGGGGTGACCG
     TATG.T..C.GCG.CA......G.T.AG.C.T.T.G.CGTCGCC....GCGGCGCAGC.GCGGTA.C.GC.G.GG.TTCTG.GGT.ACCG
```

Supplementary Figure 1. A) RNA from liver tissue was extracted and cDNA generated. PCR was performed using primers for HEV and actin as internal control. Visualization on agarose gel of amplified cDNA. **B)** cDNA was subjected to HEV specific real time PCR in a different lab (Mainz, Germany) and amplicons gel visualized. **C)** Real time RT-PCR from freshly extracted RNA (Mainz lab, Germany). Visualization on agarose gel of HEV amplicons. (Corresponding C_T values for GAPDH: case#1(FFPE block 1)=35, case#1(FFPE block 2)=38.2, case#1(FFPE block 3)=31.8, case#1(FFPE block 4)=29.9, case#3=31.1, case#2=28.8, negative control case=33.5, positive control=31.7)

Sequence analysis: **D)** Electropherograms of amplicon of patient 3 (F primer used as sequencing primer). **E)** Blast data base search revealing 182/184 (99%) identity to hepatitis E virus genomic RNA, complete genome, genotype 4, isolate: HE-JF3. **F)** Sequence comparison of amplicons of patients 3 and 4 (BioEdit Sequence Alignment Editor).
